# Fan Assisted Extraction of Volatile Carbonyl Compounds from Coffee Brews Based on the Full Evaporation Technique

**DOI:** 10.3390/foods12183389

**Published:** 2023-09-10

**Authors:** Mariana S. Aguiar, André F. S. M. R. Coelho, Paulo J. Almeida, João Rodrigo Santos

**Affiliations:** REQUIMTE/LAQV—Departamento de Química e Bioquímica, Faculdade de Ciências, Universidade do Porto, 4169-007 Porto, Portugal; up201703505@edu.fc.up.pt (M.S.A.); up201908423@edu.fc.up.pt (A.F.S.M.R.C.); pjalmeid@fc.up.pt (P.J.A.)

**Keywords:** fan assisted extraction system, full evaporation technique, volatile carbonyl compounds, 2,4-DNPH, coffee brew

## Abstract

In this work, the fan assisted extraction approach is originally exploited to determine volatile compounds in liquid samples based on the full evaporation technique. The feasibility of this strategy was firstly evaluated using model solutions containing different volatile carbonyl compounds. Different media, volumes of sample, temperatures of extraction, and times of extraction were tested. Linear regressions presenting r > 0.999, intermediate precision values < 6%, and recoveries within 76–95% were attained using a period of extraction of 10 min, a volume of sample solution of 5 µL, and a temperature of extraction of 50 °C. Analyses of brewed coffees were performed. The slopes of the calibration curves obtained using aqueous model solutions and brewed coffee samples were not significantly different. These results revealed no matrix effect under the selected experimental conditions, enabling the use of the external calibration method for quantification purposes. Twenty-four volatile carbonyl compounds were identified in brewed coffee, which elucidates the sensitivity of this approach.

## 1. Introduction

The extraction of volatile compounds from liquid samples is herein evaluated for the first time using a fan assisted extraction system based on the full evaporation technique (FET). The fan assisted extraction system was originally exploited in solid samples in the characterization of the volatile carbonyl compounds profile in raw and roasted coffee beans [1,2]. This extraction approach comprises a closed flask, wherein the sample and a container with an acceptor solution are placed. The flask features, in the inner side of its lid, an electric fan which favors, by convection, the mass transport within the gaseous phase of the flask’s headspace to the acceptor solution. Consequently, the chemical equilibrium of the volatile compounds between the sample and the headspace is shifted toward the extraction of the volatile compounds from the sample. The fan operation rapidly enriches the acceptor solution with the volatile compounds. The strategy of using the convective effect to accelerate the extraction process is the one followed by other dynamic extraction techniques [3]. This analytical system was revealed to be of simple implementation and was able to enhance volatile compound extraction either at room temperature (which is especially relevant to analyze samples that cannot be heated [1]) or when combined with sample heating procedures [2]. 

Strategies for volatile compound extraction are very extensive, with the most usual ones being headspace extraction, membrane extraction, solid phase micro-extraction, and liquid–liquid extraction [4,5]. In 1993, an alternative technique for volatile compound extraction was originally presented by Markelov et al. [6] and named as FET. These authors studied the experimental conditions, in terms of temperature of extraction and the solvent and volume of samples, for the total volatilization of the analytes prior to headspace analysis via GC-FID. It was claimed that this technique reduces the sample matrix effect typically experienced in prior volatile compound extraction strategies and maximizes the extraction ratio of the volatile compounds of the liquid sample [6]. 

FET can be performed either with static headspace analysis (FESHS) or with dynamic headspace analysis (FEDHS). In the first case, the vial containing the liquid sample is heated, and after the full evaporation of the analytes, the headspace is either sampled [7,8,9] or quantitatively transferred to the GC column [10]. In FEDHS, a purge and trap strategy is carried out; the liquid sample is generally placed inside a vial featuring a dual needle set. The vial is heated, and a stream of an inert gas flowing through the needles purges the vial’s headspace into a column filled with a sorbent material. Most commonly, a single column trap is used [9,11]. Other methodologies, referred as multi volatile method, use multiple columns containing different adsorbing materials [12,13]. To enable the full evaporation of large volumes of samples, Ochiai et al. coupled a vacuum pump to the previous strategy [13]. The FEDHS strategy was shown to be more efficient than conventional DHS or HS-SPME, as reported by Ochiai et al. [9]. The disadvantages regarding FESHS refer to the excessive inner pressure inside the sample vial as a source of operational problems [14]. In an attempt to alleviate this issue, strategies combining FET with SPME [14] or using a multiple headspace analysis [15] were reported. The main disadvantages of FEDHS, in turn, include the low sampling rate and the complex and expensive apparatus involved [16]. The usual experimental conditions in analytical methodologies based on FET are as follows: temperature above 80 °C, sample volume below 100 µL, and recovery values of the analytes typically higher than 85% [6,9,11,12,13]. The extraction periods are typically longer than 10 min [12,17,18,19,20].

The potential suitability of the fan extraction system used to perform FET is supported by the fact that the fan’s motion accelerates the sample evaporation process and the mass transport to the acceptor solution, as previously mentioned. Furthermore, this strategy contrasts from previous ones due to its simple assembly, making it feasible for use in conventional laboratories. The extraction of the analytes is performed within a closed system (in opposition to purge and trap apparatus), avoiding analyte losses and the generation of excessive pressure in the extraction system (as previously mentioned). Lastly, the herein proposed system differs from previous FET approaches by performing the extraction process into a liquid acceptor solution that can be readily injected in HPLC analytical systems. 

In this work, the feasibility of the fan assisted extraction system to promote full volatile carbonyl compound evaporation from liquid samples is evaluated with respect to temperature, period of extraction, and sample’s type of solvent and volume. Model solutions containing different volatile carbonyl compounds were tested. The acceptor solution contained a derivatizing agent, 2,4-dinitrophenylhydrazine (2,4-DNPH), to evaluate the extraction process through the analysis of the derivatized carbonyl compounds (hydrazones) via HPLC-UV/Vis.

As a case study, the characterization of the volatile carbonyl compound profile in coffee brews was investigated, and two key reasons supported this choice: (1) the foreseeable applicability of the referred derivatization reaction to this end, based on previous results with raw and roasted coffee beans [1,2], and (2) the known relevance of these compounds as key parts of the overall coffee’s volatile profile, as well as their correlation with additional important coffee characteristics, e.g., provenance, post-harvest processing method, or variety [21].

## 2. Materials and Methods

### 2.1. Reagents and Solutions

The following carbonyl compounds were used as purchased: hydroxyacetone (Alfa Aesar, Haverhill, MA, USA, L15008, 95%), furfural (Acros Organics, Hampton, NH, USA, AC181100250, ≥99%), butanone (Sigma-Aldrich, St. Louis, MO, USA, W217018-SAMPLE-K, ≥99.5%), and hexanal (Sigma-Aldrich, St. Louis, MO, USA, W255718-SAMPLE-K, ≥97%). 

For the recovery trials and for the setup of the calibration curve, a stock solution (S1) containing a mixture of the four carbonyl compounds, 2.00 × 10^−2^ mol L^−1^ in each compound, was prepared via dilution in acetonitrile. To perform the standard addition method, an additional stock solution (S2) containing a mixture of the four carbonyl compounds was prepared by diluting each compound in acetonitrile to the following concentrations: hydroxyacetone, 2.00 × 10^−1^ mol L^−1^; furfural, 2.00 × 10^−2^ mol L^−1^; butanone, 1.00 × 10^−2^ mol L^−1^; and hexanal, 4.00 × 10^−3^ mol L^−1^. 

As internal standard, a stock solution of 2-nonanone (Sigma-Aldrich, 63,969, ≥99.5%), 1.00 × 10^−2^ mol L^−1^, was prepared via dilution in acetonitrile. 

The acceptor solution was prepared in a volumetric flask of 25 mL. A total of 0.0234 g of 2,4-DNPH (TCIAD0846, >98%) was initially dissolved in 12.5 mL of acetonitrile. Afterwards, 25 µL of 2-nonanone stock solution was added. Finally, HCl solution, 40 mM, was transferred to the flask up to its calibration mark, and the resulting mixture was properly homogenized. The final concentrations in the acceptor solution were 4.0 mmol L^−1^ for the derivatizing reagent and 1.00 × 10^−5^ mol L^−1^ for the internal standard. 

For our chromatographic analysis, deionized water and acetonitrile (Fisher, Hampton, NH, USA, HPLC gradient grade, A/0627/17) were used as eluents.

The deionized water (resistivity > 18 MΩ.cm) was provided by a water purification system (Millipore Direct-Q 3UV system).

### 2.2. Fan Assisted Extraction System and Operation 

**Figure 1:**
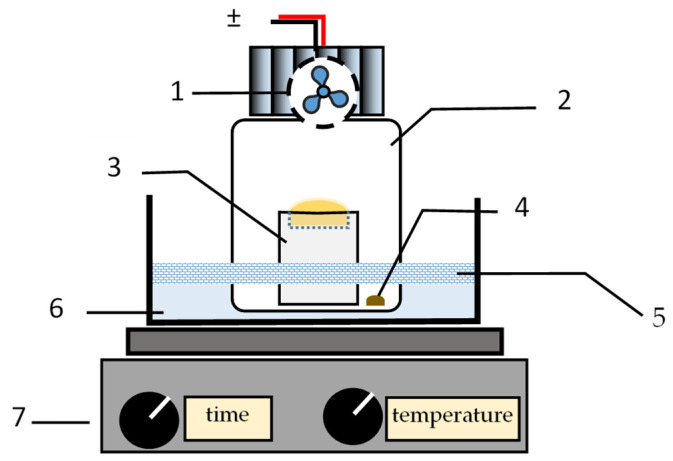
Schematic representation of the fan assisted extraction system. (1) Electric fan in the inner side of the flask’s lid; (2) glass flask; (3) PTFE reservoir containing the acceptor solution; (4) model solution/coffee brew sample; (5) polystyrene plate; (6) water bath; (7) heating plate; (±) DCV, 4.5 V.

The setup of the fan assisted extraction system apparatus was as previously described [1]. Briefly, the extraction system comprised a 100 mL flask (Schott, Mainz, Germany, GL 45, 218012458); the flask’s lid (Schott, GL 45; Polytetrafluoroethylene (PTFE) septum—Duran Wheaton Kimble, Mainz, Germany, GL 45, ref. 292482806), which featured an electric fan (Coolerguys, Kirkland, WA, USA, 25 × 25 × 10 mm, IP67, 4.5 V) in its inner side; and a PTFE reservoir (a cylinder of 43 mm h × 20 mm Ø presenting a cavity with a capacity of ca. 500 µL in one of its bases) (Figure 1).

The experimental procedure followed to perform the extraction of the volatile compounds from the liquid samples was as follows: A PTFE cylinder was firstly placed inside the flask, and the acceptor solution was transferred into the cylinder’s cavity. Thereafter, the liquid sample was transferred to the bottom’s flask. The flask was closed with a lid and placed in a water bath so that only the bottom’s flask was immersed. A plate of polystyrene (presenting a hole of the flask’s diameter) was placed to minimize the heating of the part of the flask that was not immersed. The electric fan was kept on during a defined time of extraction. At the end of the extraction trial, the fan was turned off, and the flask was removed from the water bath. Lastly, the flask was opened, and the acceptor solution transferred into a vial to be analyzed using the HPLC system. 

At the end of each extraction trial, the flask and the PTFE cylinder were washed with a water/acetonitrile solution, 1:1 (*v*/*v*), and dried before further utilization. In parallel, the fan was left on for at least 10 min between trials to avoid carryover occurrence. 

### 2.3. Recovery Trials with Model Solutions

Through the proper dilution of the stock solution S1, model solutions of the carbonyl compounds, 2.00 × 10^−4^ mol L^−1^, were prepared in three different media, namely, water; water/acetonitrile, 1:1 (*v*/*v*); and acetonitrile. Recovery trials for each of the three model solutions were then conducted, each with different temperature conditions, sample volume, and times of extraction. The temperatures tested were 30 °C and 50 °C; the sample volumes tested were 5.0, 10.0, 20.0, and 30.0 µL, and the periods of extraction evaluated were 5, 10, and 20 min. The volume of acceptor solution was 400 µL. The analytical signal respective to 100% recovery was obtained by mixing the referred volumes of sample directly with 400 µL of the acceptor solution (bypassing the extraction procedure). 

The recovery trials were conducted using the fan assisted extraction system (operated as previously mentioned) (Section 2.2). In order to assess the fan operation impact upon the extraction process, the recovery trials were also conducted with the fan turned off.

### 2.4. Precision Trial and Calibration Curves Using Model Solutions of Volatile Carbonyl Compounds

An evaluation of the precision of the experimental procedure was conducted by performing quadruplicate extractions of an aqueous model solution containing a mixture of the studied volatile carbonyl compounds (2.00 × 10^−4^ mol L^−1^), for three consecutive days. The relevant experimental conditions used were as follows: temperature of extraction, 50 °C; sample volume, 5 µL; period of extraction, 10 min; and volume of acceptor solution, 400 µL.

The calibration curves were set up using five aqueous diluted solutions of stock solution S1 within the interval of 2.00 × 10^−5^ and 2.00 × 10^−4^ mol L^−1^. The relevant experimental conditions used were the same as those mentioned in the previous paragraph. The calibration curves were performed in duplicate.

### 2.5. Extraction Condition Studies of Carbonyl Volatile Compounds in Coffee Brew

Two studies were performed. The initial study used a fortified coffee brew (1.00 mL of coffee brew + 10 µL of solution S2) to characterize the volatile carbonyl compound extraction during different periods of extraction, namely 5, 10, 15, and 20 min. Based on previous results using model solutions, the relevant experimental conditions used were as follows: sample volume, 5 µL; acceptor solution volume, 400 µL; and water bath temperature, 50 °C. These extraction trials were performed in duplicate with fan-on and fan-off conditions.

In the second study, the coffee matrix effect was evaluated by performing the standard addition method and the external calibration method in parallel. In the standard addition method, brewed coffee was fortified at four different levels via appropriate additions of stock solution S2 to enable increments of ca. 50%, 100%, 150%, and 200% for each carbonyl compound. The external calibration method was set up using aqueous standard solutions containing the same increments as for the standard addition method procedure.

The coffee brews used were prepared as described below (Section 2.6).

To transfer 5 µL of brewed coffee into the flask of the fan assisted extraction system, a 10 µL capacity glass syringe (Hamilton, Reno, NV, USA, ref. 549-0223) was used.

### 2.6. Coffee Brewing

Coffee brews were prepared according to the most conventional brewing procedures, i.e., using decoction, infusion, and espresso methods [22]. A single commercial grinded coffee blend was used in the preparation of the brews. Therefore, the sample was considered homogeneous in its physical and chemical composition. For each extraction procedure, a sample mass of 5.80 g was used. The extraction procedures were performed as follows: in the decoction method, deionized water (40 mL) was heated until boiling. Afterwards, the heating was stopped, and the coffee was added. The mixture was left under gentle mixing for 2 min before decantation. The infusion method was performed as follows: the coffee was transferred onto filter paper previously placed in a glass funnel. A total of 40 mL of boiled deionized water was then slowly transferred and allowed to drip. For the espresso extraction method, the coffee was poured into a capsule compatible with a Nespresso^®^ machine. Deionized water was used, and the extraction volume was pre-set to ca. 40 mL. 

At the end of the brewing processes, the final volume of the coffee extracts was adjusted with deionized water to 40.0 g by weighing. Next, the resulting solution was homogenized and finally transferred into a closed flask until reaching room temperature.

### 2.7. Chromatographic Analyses (HPLC-UV/Vis and HPLC-DAD-MS/MS)

The chromatographic analyses of all prepared solutions and extracts were performed using an HPLC from Jasco that consisted of a quaternary piston pump (PU-2089 Plus), an autosampler (AS-2055 Plus), and an UV/Vis detector (UV-2070 Plus). The data treatment was performed using Chrompass software (Jasco, Tokyo, Japan, v.1.8.6.1). The elution conditions, being based on previous work, were as follows [21]: the stationary phase was a column Gemini C_18_ from Phenomenex (150 mm × 4.6 mm, 3 μm), and the elution was performed in gradient mode using water and acetonitrile (0 min: 50% acetonitrile, 50% water; 20 min: 65% of acetonitrile, 35% water; 45 min: 100% of acetonitrile; 50 min: 50% of acetonitrile, 50% water; and 55 min: 50% of acetonitrile, 50% water); the mobile phase flow rate was 0.45 mL min^−1^. The volume of the injected sample was 25 µL, and the wavelength of detection was set at 360 nm.

For the identification of carbonyl compounds, the extracts obtained using the fan assisted extraction procedure were analyzed using an HPLC-DAD-MS/MS system, model LTQ XL, from Thermo Scientific (Waltham, MA, USA). The same elution conditions as above-mentioned were used. The settings of the mass spectrum detector (see Supplementary Data S1) were adjusted via proper tuning with the hydrazones of the carbonyl compounds studied. 

## 3. Results and Discussion

### 3.1. Preliminary Trials

A preliminary trial to study the period required to fully evaporate a liquid sample using the fan assisted extraction system was conducted. Different sample volumes prepared in different media were transferred into the flask, and the period until full evaporation was measured using different water bath temperatures (30 and 50 °C) and fan operation statuses (on and off) (Table 1).

As expected, shorter periods for full sample evaporation were observed for the smaller volumes tested (for samples prepared via increasing acetonitrile content under fan assisted conditions). The fan on effect was more pronounced at mild conditions (30 °C) as greater differences in the periods required until full sample evaporation were observed compared to the fan off conditions. 

The tests performed with sample volumes higher than 10 µL enabled the visualization of condensation spots in the upper region of the flask. Furthermore, the impact of the fan’s operation upon the mass transfer process inside the flask was well evident as the condensation spots changed their location during the extraction trial. During these trials, different mass losses of acceptor solution depending on the experimental conditions used were also noticed. Based on these observations, an internal standard (2-nonanone) was added into the acceptor solution in subsequent tests. For data treatment purposes, the internal standard method was followed for each studied compound. 

### 3.2. Recovery Values for Volatile Carbonyl Compounds in Model Solutions

In this study, carbonyl compounds of different chemical structures were considered, namely, an hydroxyketone (hydroxyacetone), a cyclic carbonyl compound (furfural), a *n*-ketone (butanone), and a *n*-aldehyde (hexanal). These compounds were already identified by HPLC-UV after derivatization with 2,4-DNPH in coffee samples [1,2,21], the case study selected later on, justifying the suitability of this option in the present work.

**Figure 2:**
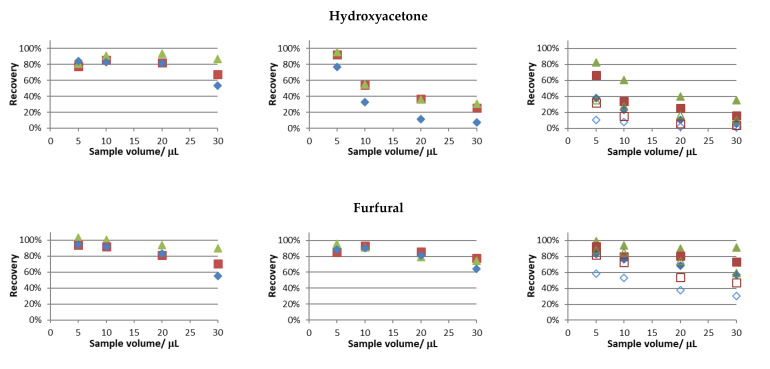

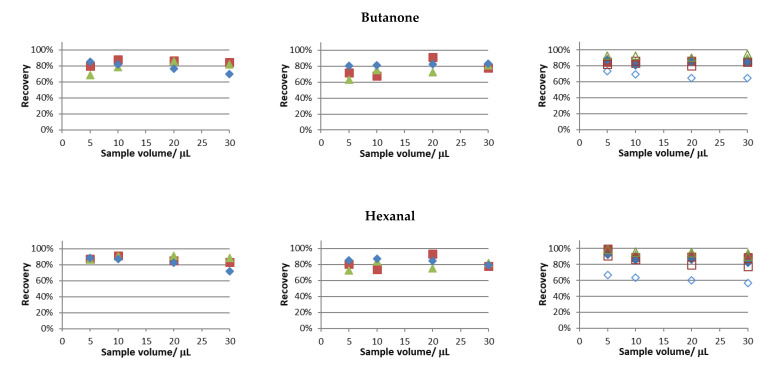
Recovery values obtained for a model solution prepared in acetonitrile (**left-side column**), water/acetonitrile, 1:1 (*v*/*v*) (**middle column**), and water (**right-side column**) at 50 °C. Extraction periods (♦) 5 min; (■) 10 min; (▲) 20 min. Unfilled symbols in right column refer to recovery values with fan off. RSD < 5.8% (n = 2).

The recovery trials were performed using the experimental conditions tested in the previous section, and the obtained results are presented in Figure 2.

Similar recovery values for butanone and hexanal were attained in all studies performed. Notably, considering this behavior at 30 °C and 50 °C and the fact that only in the latter condition full sample evaporation was attained (see Supplementary Data S3), full sample evaporation is not a sine qua non condition for near full analyte recovery. 

In this set of studies, the main differences were observed for hydroxyacetone and furfural, and the following general observations were made: the highest water bath temperature, the lower sample volumes, and the higher periods of extraction provided superior recoveries of the studied analytes. This was an expected result, considering that sample evaporation is faster for the two parameters first mentioned and higher extraction periods provide more time for the volatile compounds to reach the acceptor solution. Furthermore, in aqueous solutions, the recoveries of these compounds were the lowest among the tested compounds. The values found regarding Henry’s law solubility constants in water [23] support the results obtained. 

The differences in the extraction profiles of the studied compounds can be attributed to different adsorption phenomena in the different surfaces of the experimental setup and, to the dissolution of the analytes in the condensation spots (especially when larger volumes of sample were used).

In order to further evaluate the impact of fan operation, the recovery trial involving the use of a mixture of carbonyl compounds in aqueous media and a temperature condition of 50 °C was replicated with the fan off (the results are superimposed in the left-side column of Figure 2). Clearly, this led to the lowest recoveries achieved, especially when short extraction periods and high sample volumes were tested. This pattern is more evident for furfural, and mainly, hydroxyacetone, the ones less recovered without fan assisted conditions. The results for these compounds support the proof of concept of using the fan assisted extraction approach, i.e., within the different extraction periods and sample volumes tested, the recovery values were always higher under fan assisted conditions. 

Based on the results achieved to this point, further trials were performed according to the following experimental conditions: sample volume, 5 µL; period of extraction, 10 min; temperature of extraction, 50 °C; and sample media, water. In fact, recoveries above 80% were observed for all studied carbonyl compounds, except for hydroxyacetone, whose recovery was markedly correlated with increasing acetonitrile content in the sample media. 

### 3.3. Precision Trials and Calibration Curves Using Model Solutions

Using the selected experimental conditions (Section 2.4), typical results of repeatability and intermediate precision trials, in terms of RSD values, for each of the carbonyl compounds studied are presented in Table 2. 

The RSD values for repeatability and intermediate precision trials (lower than 6%) were considered acceptable, enabling the use of the selected experimental conditions in further trials. 

In Table 3, the parameters of linear regression for each of the studied carbonyl compounds are presented. The results showed a linear proportionality between the chromatographic peak area of each carbonyl compound extracted and its respective concentration in the sample solution. The mean recoveries of the volatile carbonyl compounds studied were within 76% for hydroxyacetone and 95% for hexanal. These figures of merit are similar to the ones reported in previous analytical strategies to perform the full evaporation technique (which have already been summarized in the introduction section). These results verify the applicability of the developed approach to quantify volatile compounds in aqueous samples. 

### 3.4. Case Study: Extraction of Volatile Carbonyl Compounds from Coffee Extracts

**Figure 3:**
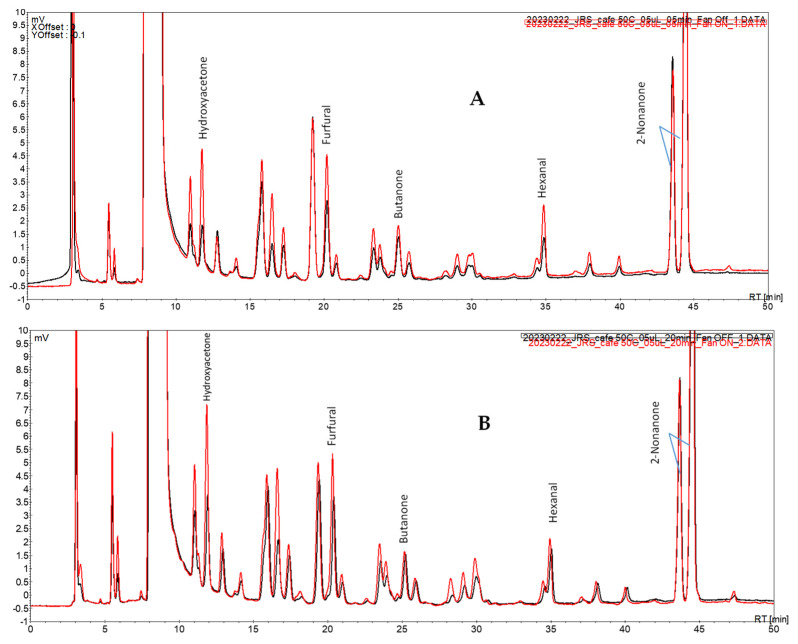
Chromatograms of a spiked coffee brew sample using the fan assisted extraction system under different experimental conditions: (**A**) 5 min; (**B**) 20 min; (▬▬) fan on; (▬▬) fan off.

**Figure 4:**
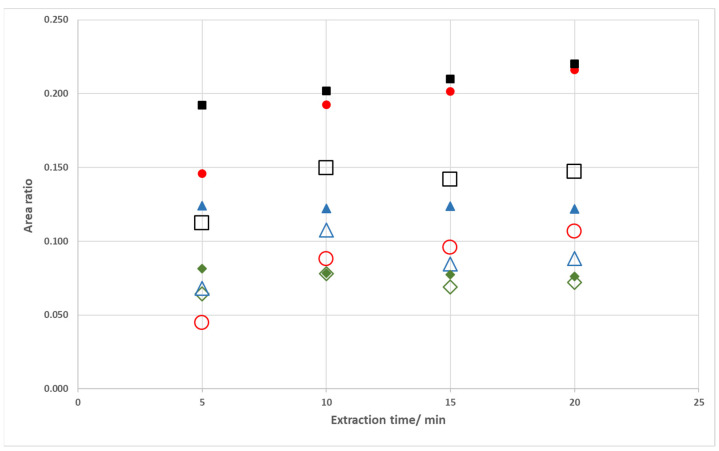
Area ratios of the chromatographic peaks of carbonyl compounds in a spiked coffee brew for different extraction periods using the fan assisted extraction system. (●) hydroxyacetone; (■) furfural; (◆) butanone; (▲) hexanal. Unfilled symbols are representative of the extraction trials performed with the fan off.

The initial studies with a spiked coffee brew (Section 2.5) focused on the impact of the fan assisted conditions upon the extraction of the volatile carbonyl compounds for different periods of extraction. The chromatograms obtained after periods of 5 and 20 min and the extraction profile of each of the studied carbonyl compounds are presented in Figure 3 and Figure 4, respectively. 

Under fan assisted conditions (vs. fan off conditions) at all periods assayed, the overall profile showed peaks of higher intensity that were more noticeable for the peaks at Rt 10.8, 11.8, 16.9, and 28.2 min (Figure 3).

Among the extraction periods tested, one observed a progressive signal increase for hydroxyacetone and furfural and a plateau profile for butanone and hexanal (Figure 4). This is a similar behavior to the one previously depicted using model solutions. Furthermore, the proof of concept of this approach was again observed in this case study: the extraction of the volatile carbonyl compounds under fan assisted conditions occurred to an extent not attainable by solely increasing the extraction period. 

Based on these results, a 10 min extraction period was further evaluated with respect to repeatability since this period enabled a reasonable sample analysis throughput without compromising the sensitivity of this methodology. Using this extraction period, RSD values were lower than 5.2% (*n* = 3) for all peaks presenting area values higher than 0.2 mV.min.

The standard addition method was applied to coffee brews prepared via different brewing techniques (as described in Section 2.5). The obtained results enabled us to establish a linear calibration curve for each of the carbonyl compounds added. To evaluate the coffee matrix effect, the slope of each linear regression obtained via the standard addition method was statistically compared with the slope obtained using aqueous model solutions. To this end, Student’s *t*-test was performed after assessing the standard deviation of y-residuals (s_y/x_) through the F-test [24]. These results are presented in Table 4 and enabled us to conclude that the null hypothesis for the F-test and t-test is retained as either the differences on the variances and slopes are not statistically significant. These results support the potential of combining the fan assisted extraction method and the external calibration method to perform the full evaporation technique and quantification of volatile analytes in coffee brews.

### 3.5. Profile of Carbonyl Volatile Compounds in Coffee Brews from Different Brewing Techniques

**Figure 5:**
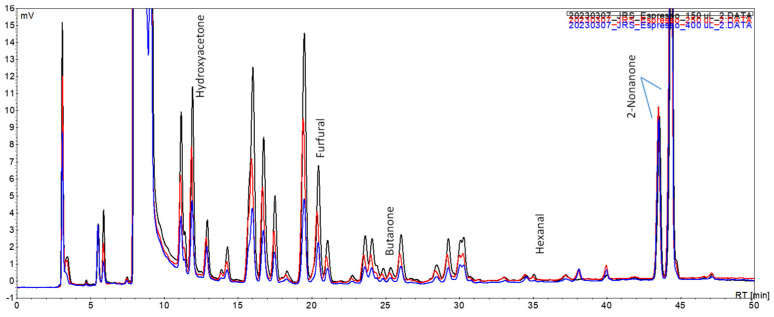
Chromatograms of different volumes of acceptor solution after the fan assisted extraction of an espresso coffee sample. (▬▬) 150 µL, (▬▬) 250 µL, and (▬▬) 400 µL.

In order to increase the chromatographic peaks’ intensity and thereafter improve the HPLC-DAD-MS/MS data in terms of product and precursor ion formation for carbonyl compound identification, a preliminary trial was conducted by testing different volumes of acceptor solution, namely, 400 µL, 250 µL, and 150 µL (Figure 5).

A proportional increase in the intensity of the peaks was observed when lowering the volume of the acceptor solution. For the identification of the volatile carbonyl compounds of coffees from different brewing techniques, a volume of 150 µL was selected. As well as fitting the purpose of improving the aforementioned HPLC-DAD-MS/MS data, this option showed that increasing the enrichment factor by reducing the acceptor solution volume is a relevant potentiality of this approach to be further exploited, if one considers the usual volumes of sample injected in, e.g., GC systems.

**Figure 6:**
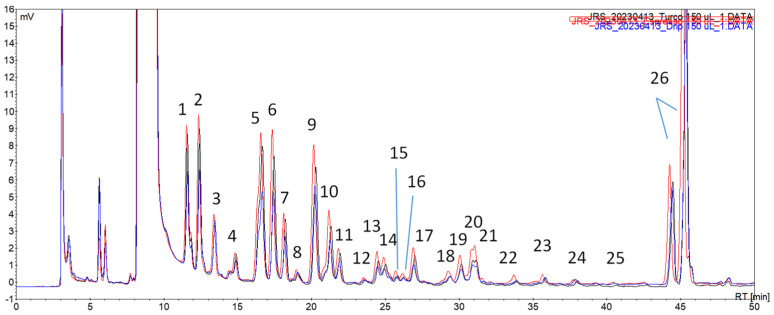
Chromatograms of extracts from coffees prepared according to different brewing methods. (▬▬) decoction; (▬▬) infusion; (▬▬) espresso. The numbered peaks are identified in Table 5.

The following profiles were then obtained for a commercial coffee blend using conventional brewing techniques, i.e., decoction, infusion, and espresso (Figure 6).

The extracts from each coffee brew were analyzed via HPLC-DAD-MS/MS, and the compiled data is presented in Table 5. Only the chromatographic peaks of areas higher than 0.2 mV.min and presenting a discernible precursor ion from the baseline signals were considered. The identification of the compounds was performed based on the respective precursor and product ions, the maximum wavelength, and retention time already observed in former works [1,2,21]. 

The profiles of the volatile carbonyl compounds for the different brewing methods were similar in terms of the number of peaks observed. The main difference was the higher intensity profile observed for the espresso brew, suggesting the higher extraction efficiency of this brewing procedure. This was an expected result based on similar findings, which compared the impact of espresso vs. moka and drip coffee brewing techniques [25], espresso vs. cezve and drip brew [26], and espresso vs. drip brew [27].

The sensitivity of the developed extraction procedure, using solely 5 µL of sample volume, was noteworthy. Indeed, this approach enabled us to identify nearly all of the volatile carbonyl compounds in the green and roasted coffee beans, as previously reported in earlier works, using the same derivatization reaction [1,2]. The analytical application of this derivatization reaction to identify carbonyl compounds in brewed coffee was not found described.

## 4. Conclusions

This work presented an original approach to determine volatile compounds in liquid samples using a fan assisted extraction system and exploiting the FET concept. This approach distinguishes itself from other reported strategies by combining the following features: FET is assisted by an electric fan; the extracted compounds are collected into a liquid acceptor solution; the apparatus (based solely on a flask featuring an inner electric fan and a container for the acceptor solution) can be easily replicated in conventional laboratories and has the advantages of low operation and implementation costs. The recoveries of volatile compounds were within 76 and 95%. These figures of merit were similar to that of other previously reported analytical systems used to perform FET. Furthermore, the approach herein evaluated can be performed at lower temperatures (50 °C), uses smaller sample volumes (5 µL), and can be performed for shorter periods (10 min) compared to other FET methodologies, therefore highlighting the convenience of this approach. 

The results obtained with the brewed coffee samples highlighted the positive impact of the fan motion on the recovery rate of the volatile compounds (values not attainable solely using longer extraction periods). No coffee matrix effect was observed for the compounds studied, thereby validating the possibility of using this analytical approach in the quantification of other volatile carbonyl compounds through the external calibration method. 

Lastly, this approach was able to identify twenty-four volatile carbonyl compounds, exhibiting similar performance compared to previous works featuring raw and roasted whole coffee beans and the use of the same derivatization reaction. This methodology is complementary to previous works as it illustrates that different coffee matrices (i.e., raw, roasted, and brewed roasted coffee) can be analyzed with the aim of identifying volatile carbonyl compounds using a unique derivatization reaction.

## Figures and Tables

**Table 1:** Mean times measured until full aqueous sample evaporation under different experimental conditions using the fan assisted extraction system (*n* = 2) *.

Sample Volume/µL	Water Bath Temperature			
	30 °C		50 °C	
	Fan Off	Fan On	Fan Off	Fan On
5.00	>20 min	12 min 20 s	5 min 46 s	3 min 30 s
10.00	>20 min	>20 min	8 min 42 s	6 min 24 s
20.00	>20 min	>20 min	11 min 34 s	8 min 37 s
30.00	>20 min	>20 min	16 min 10 s	11 min 43 s

* full data available in Supplementary Data S2.

**Table 2:** RSD values calculated for the repeatability and intermediate precision trials of volatile carbonyl compounds using the fan assisted extraction system.

Carbonyl Compound	Repeatability– 1 Day (n = 4)	Intermediate Precision– 3 Days (n = 12)
Hydroxyacetone	4.3%	5.6%
Furfural	2.3%	5.9%
Butanone	0.6%	2.3%
Hexanal	1.2%	2.4%

**Table 3:** Parameters of the calibration curves obtained for different volatile carbonyl compounds using the fan assisted extraction system.

Carbonyl Compounds	Slope/ (10^−2^ mol L^−1^)	Intercept/10^3^	r	l.o.d./ (10^5^ mol L^−1^)	Mean Recovery/%
Hydroxyacetone	1.61 ± 0.04	0.133 ± 0.447	0.9999	0.311	76.5
Furfural	8.26 ± 0.56	3.59 ± 6.94	0.9993	0.942	91.4
Butanone	10.6 ± 0.1	5.92 ± 8.13	0.9994	0.860	86.5
Hexanal	12.4 ± 1.1	4.46 ± 13.1	0.9989	1.19	94.3

**Table 4:** Values of Student’s *t*-test and F-test calculated for the slope values obtained in the standard addition method for different brewed coffee samples vs. model solutions.

Carbonyl Compound	Espresso Method			Infusion Method			Decoction Method		
	r	F-Test	*t*-Test	r	F-Test	*t*-Test	r	F-Test	*t*-Test
Hydroxyacetone	0.991	3.77	2.10	0.999	1.45	0.34	0.996	1.58	1.90
Furfural	0.999	1.28	0.15	1.000	1.79	1.10	0.999	1.18	0.80
Butanone	0.998	1.46	0.83	1.000	5.64	0.35	0.998	3.38	0.62
Hexanal	0.999	2.72	1.50	0.999	1.77	0.86	0.996	1.13	0.55

t critic = 2.45 (95%, ν = 6); F critic = 15.44 (95%, ν_1_ and ν_2_ = 3).

**Table 5:** Data from HPLC-DAD-MS/MS analyses of coffee extracts obtained using the fan assisted extraction system.

Peak No	Compound	λ/nm	MS	MS2	MS3	Std/Ref
1	Acetoin	364	267	177, 179, 152; 120	147	Std
2	Hydroxyacetone (*E*-isomer)	370	253	177; 179, 152	151	Std
3	Formaldehyde	352	209	163; 120; 151; 179	133	Std
4	2-Hydroxy-3-pentanone	361	281	177; 179; 152; 182	147	Ref *
5	Acetaldehyde	364	223	179; 163; 151	151	Std
6	C_5_H_8_O_3_	361	475	277; 295; 179; 167	230; 181	Ref *
7	2-Methyloxolan-3-one	361	279	191; 179; 249; 151	164; 173; 159	Std
8	2-Hydroxy-3-pentanone	361	281	152; 191; 179	122	Ref *
9	Propanone	367	237	151; 179; 207	121	Std
10	Furfural	388	275	228; 163; 179	170	Std
11	Propanal	361	237	163; 179; 192	105	Std
12	Furfural	382	275	228; 163; 179	170	Std
13	5-Methylfurfural	400	289	242; 163; 179	200; 214	Std
14	2-Acetylfuran	<Abs	289	259; 242; 163; 151	217; 184	Std
15	1-(1H-pyrrol-1-yl)-2-propanone	<Abs	302	179; 272; 151	151	Ref *
16	Butanone	367	251	221; 179; 151	-	Std
17	Butanal	364	251	163; 179; 205	-	Std
18	Benzaldehyde	382	285	163; 238; 121; 179	105; 133	Std
19	5-Methylfurfural	388	289	242; 163; 179	200; 214	Std
20	2-Methylbutanal	367	265	163; 179; 235; 152	105	Std
21	Pentanal	367	265	163; 179; 219; 235	105	Std
22	Methylglyoxal	<Abs	431	182; 251	152; 122	Std
23	Hexanal	364	279	163; 179; 152; 205	105	Std
24	Diacetyl	379	445	182; 399; 364; 265	152; 122	Std
25	2,3-Pentanodione	400	459	182; 413; 412; 279	152; 122	Std
26	2-Nonanone **	367	321	291; 152; 179	152; 122	Std

* previous discussion in [21]; ** added as internal standard.

## Data Availability

Data is contained within the article or Supplementary Material.

## Supplementary Materials

The following supporting information can be downloaded at: https://www.mdpi.com/article/10.3390/foods12183389/s1, Supplementary Data S1: Parameter values of the single-quadrupole ion trap mass spectrometer (Thermo Scientific LTQ XL); Supplementary Data S2: Table S1—Mean times measured until full sample evaporation under different experimental conditions using the fan assisted extraction system (n = 2); Supplementary Data S3: Figure S1—Recovery values obtained for a model solution prepared in acetonitrile (left-side column), water/acetonitrile, 1:1 (*v*/*v*) (middle column), and water (right-side column) at 30 °C.
