# Supplementary material for: Fan Assisted Extraction of Volatile Carbonyl Compounds from Coffee Brews Based on the Full Evaporation Technique
Source: Foods. 2023 Sep 10;12(18):3389. doi: 10.3390/foods12183389 (PMC10528458; doi:10.3390/foods12183389)
Supplement: Supplementary file 1 [file foods-12-03389-s001.zip › Supplementary Data S1_jrs.pdf]

Supplementary Data S1

Fan Assisted Extraction of Volatile Carbonyl Compounds from Coffee Brews Based on the Full Evaporation Technique

Mariana S. Aguiar, André F. S. M. R. Coelho, Paulo J. Almeida, João Rodrigo Santos

REQUIMTE/LAQV - Departamento de Química e Bioquímica, Faculdade de Ciências, Universidade do Porto, Porto, Portugal

Parameter values of the single quadrupole ion-trap mass spectrometer (Thermo Scientific LTQ XL)

HESI Source
Capillary temperature: 300 °C
Source Heater temperature: 350 °C
Sheath gas, N2, (arbitrary units): 40
Auxiliary gas flow (arbitrary units): 15
Zoom AGC target: 3000
Full AGC target: 10000
SIM AGC target: 10000
MSn AGC target: 10000
Source Voltage (kV): 3.20
Source Current (μA): 100.00
Capillary Voltage (V): -30.00
Tube Lens Voltage (V): -65
Multipole RF Amplifier (Vp-p): 400
Multipole 00 Offset (V): 4.50
Lens 0 (V): 5.0
Multipole 0 Offset (V): 5.50
Lens 1 (V): 30
Multipole 1 Offset (V): 10
Front Lens (V): 5.5
Zoom micro scans: 1
Zoom Max ion time (ms): 10.00
Full micro scans: 1
Full Max Ion time (ms): 10.00
MSn micro scans: 1
MSn Max Ion time (ms): 10.00
