# Supplementary material for: Fan Assisted Extraction of Volatile Carbonyl Compounds from Coffee Brews Based on the Full Evaporation Technique
Source: Foods. 2023 Sep 10;12(18):3389. doi: 10.3390/foods12183389 (PMC10528458; doi:10.3390/foods12183389)
Supplement: Supplementary file 1 [file foods-12-03389-s001.zip › Supplementary Data S2_jrs.pdf]

Supplementary Data S2

Fan Assisted Extraction of Volatile Carbonyl Compounds from Coffee Brews Based on the Full Evaporation Technique

Mariana S. Aguiar, André F. S. M. R. Coelho, Paulo J. Almeida, João Rodrigo Santos

REQUIMTE/LAQV - Departamento de Química e Bioquímica, Faculdade de Ciências, Universidade do Porto, Porto, Portugal

Table S1. Mean times measured until full sample evaporation under different experimental conditions using the fan assisted extraction system (n = 2)

Sample Volume/ μL	Acetonitrile Media				Water: Acetonitrile Media, 50:50, v/v				Water Media			
	30 °C*		50 °C*		30 °C*		50 °C*		30 °C*		50 °C*	
	Fan Off	Fan On	Fan Off	Fan On	Fan Off	Fan On	Fan Off	Fan On	Fan Off	Fan On	Fan Off	Fan On
5.00	1 min 26 s	1 min 1 s	0 min 38 s	0 min 28 s	13 min 05 s	7 min 55 s	2 min 0 s	1 min 31 s	> 20 min	12 min 20 s	5 min 46 s	3 min 30 s
10.00	2 min 26 s	1 min 18 s	1 min 01 s	0 min 44 s	> 20 min	11 min 59 s	3 min 49 s	2 min 35 s	> 20 min	> 20 min	8 min 42 s	6 min 24 s
20.00	4 min 40 s	1 min 47 s	1 min 21 s	0 min 56 s	> 20 min	16 min 48 s	5 min 57 s	3 min 55 s	> 20 min	> 20 min	11 min 34 s	8 min 37 s
30.00	12 min 8 s	4 min 12 s	2 min 22 s	1 min 17 s	> 20 min	> 20 min	8 min 52 s	5 min 10 s	> 20 min	> 20 min	16 min 10 s	11 min 43 s

* temperature of the water bath
