# Supplementary material for: Fan Assisted Extraction of Volatile Carbonyl Compounds from Coffee Brews Based on the Full Evaporation Technique
Source: Foods. 2023 Sep 10;12(18):3389. doi: 10.3390/foods12183389 (PMC10528458; doi:10.3390/foods12183389)
Supplement: Supplementary file 1 [file foods-12-03389-s001.zip › Supplementary Data S3_jrs_2.pdf]

Supplementary Data S3

Fan Assisted Extraction of Volatile Carbonyl Compounds from Coffee Brews Based on the Full Evaporation Technique

Mariana S. Aguiar, André F. S. M. R. Coelho, Paulo J. Almeida, João Rodrigo Santos

REQUIMTE/LAQV - Departamento de Química e Bioquímica, Faculdade de Ciências, Universidade do Porto, Porto, Portugal

Hydroxyacetone

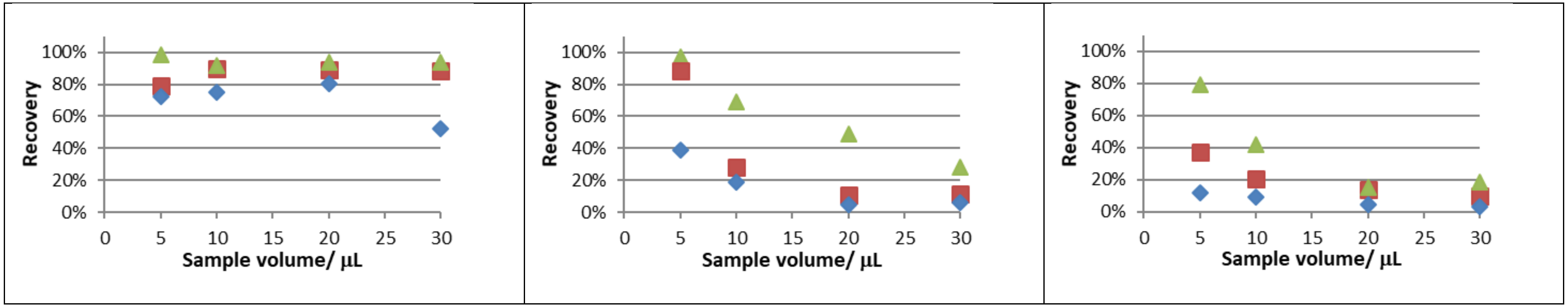

Furfural

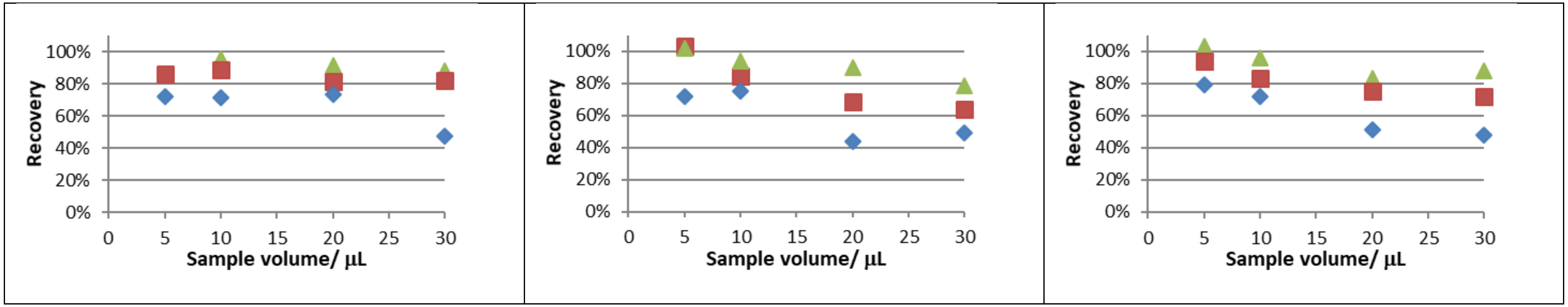

Figure S1. *Cont.*

Butanone

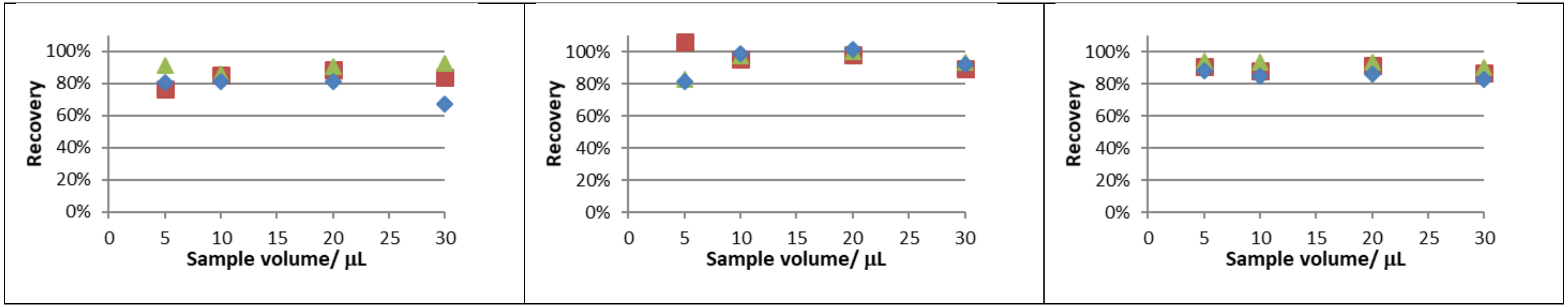

Hexanal

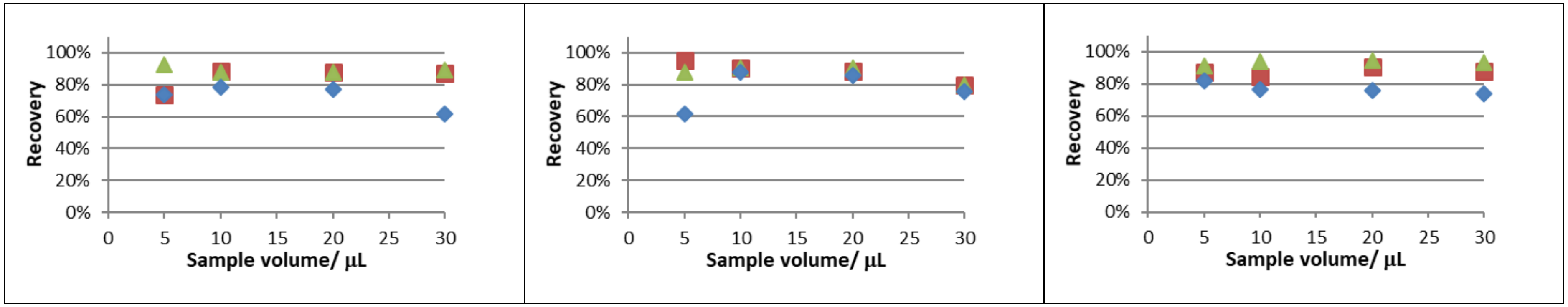

Figure S1. Recovery values obtained for a model solution prepared in acetonitrile (left side column), water:acetonitrile, 1:1 (v/v) (middle column) and water (right side column) at 30 °C. Extraction periods (◆) 5 min; (■) 10 min; (▲) 20 min. RSD < 5.5% (n = 2).
